# A functionalized method for assessing the risk of adverse events in patients with pulmonary embolism

**DOI:** 10.3389/fmed.2025.1701432

**Published:** 2025-12-19

**Authors:** Keyu Sun, Long Yu, Zichen Xie, Haisu Lu, Shengzhang Wang

**Affiliations:** 1 Institute of Biomechanics, College of Biomedical Engineering, Fudan University, Shanghai, China; 2 Emergency Department, Minhang Hospital, Fudan University, Shanghai, China; 3 Transvascular Implantation Devices Research Institute, Hangzhou, China; 4 School of Public Health, Zhejiang University, Hangzhou, China

**Keywords:** function analysis, physiological parameters, pulmonary embolism, risk assessment, weight coefficient

## Abstract

**Background:**

Pulmonary embolism (PE) is a common and potentially fatal condition in emergency medicine, and accurate early risk stratification is critical for guiding clinical management. Existing prognostic models, such as the Pulmonary Embolism Severity Index (PESI) and PERFORM, rely on discrete scoring systems and subjective variables, which may introduce bias and limit predictive precision.

**Methods:**

In this study, we propose CON-PERFORM, a novel functional prognostic model that incorporates three readily available objective parameters: age, heart rate, and arterial partial pressure of oxygen (PaO_2_). Weighting coefficients were determined through functional optimization rather than empirical cutoffs, yielding a continuous risk score designed to improve accuracy and threshold stability. A retrospective cohort of 559 objectively confirmed PE patients (373 in the training set, 186 in the validation set) was used to construct and evaluate the model, and an independent prospective cohort of 128 patients was collected for further validation.

**Results:**

Compared with the PERFORM method, CON-PERFORM demonstrated superior diagnostic performance, with higher area under the curve (AUC), accuracy, and specificity, while maintaining comparable sensitivity. In the training cohort, CON-PERFORM achieved an AUC of 0.841 versus 0.793 for PERFORM, with consistent improvements observed in both the validation and prospective cohorts. Moreover, CON-PERFORM effectively stratified patients into high- and low-risk groups, which displayed distinct survival and discharge patterns over 30 days.

**Conclusion:**

In conclusion, CON-PERFORM provides a simple, objective, and robust tool for individualized risk assessment in PE. Its improved diagnostic performance and stability across cohorts highlight its potential for clinical application and resource optimization in emergency care.

## 1 Introduction

Pulmonary embolism (PE) is a common condition in emergency departments and is associated with high rates of mortality and morbidity, necessitating timely and accurate treatment. Fatal outcomes typically occur within weeks of diagnosis (1, 2). The short-term mortality of PE varies markedly: while the mortality rate is less than 2% in many patients with non-severe PE, it can exceed 95% among those who experience cardiopulmonary arrest (3–6). Accordingly, accurate prediction of prognosis in acute PE carries substantial clinical value.

Several prognostic models have been developed for acute PE. Among them, the Pulmonary Embolism Severity Index (PESI) and its simplified version (sPESI)—which integrate PE severity with comorbidities—are the most widely validated clinical scoring systems (7–10). However, these models rely heavily on subjective variables, such as a history of malignancy or chronic cardiopulmonary disease. When assessed through patient interviews, such variables may introduce bias and potentially lead to misclassification of mortality risk (11, 12). Computed tomography pulmonary angiography (CTPA) remains the diagnostic gold standard for PE. Yet, in practice, patient identification often relies solely on International Classification of Diseases (ICD) codes. This approach risks including individuals with PE-like symptoms but without confirmed PE, limiting the ability to accurately predict 30-day mortality in CTPA-confirmed patients (13, 14).

Given these limitations, there is a clear need for a prognostic model that is objective, accurate, and straightforward to apply in clinical settings, thereby supporting risk stratification and treatment decisions. For instance, low-risk patients may benefit from early discharge or outpatient management, whereas high-risk patients require closer monitoring and aggressive interventions (15–18). To address this, Yu et al. proposed a simple prognostic model that estimates 30-day mortality based on objective parameters such as age, heart rate, and partial pressure of oxygen (PaO_2_), independent of past medical history or subjective assessments (19). Clinical validation demonstrated superior predictive performance compared with traditional models, while relying exclusively on objective measurements. Nonetheless, two major limitations remain: (a) the parameter cutoffs are largely experience-driven and thus not fully objective; and (b) the use of discrete thresholds introduces discontinuities that may impair risk estimation accuracy.

To overcome these challenges, the present study establishes a prognostic model for PE-related adverse outcomes based on age, heart rate, and PaO_2_. By employing functional analysis, we derive weight coefficients that are free from subjective selection, and validate the model using clinical datasets to confirm its predictive utility.

## 2 Study participants and methods

### 2.1 Participants

**Figure 1:**
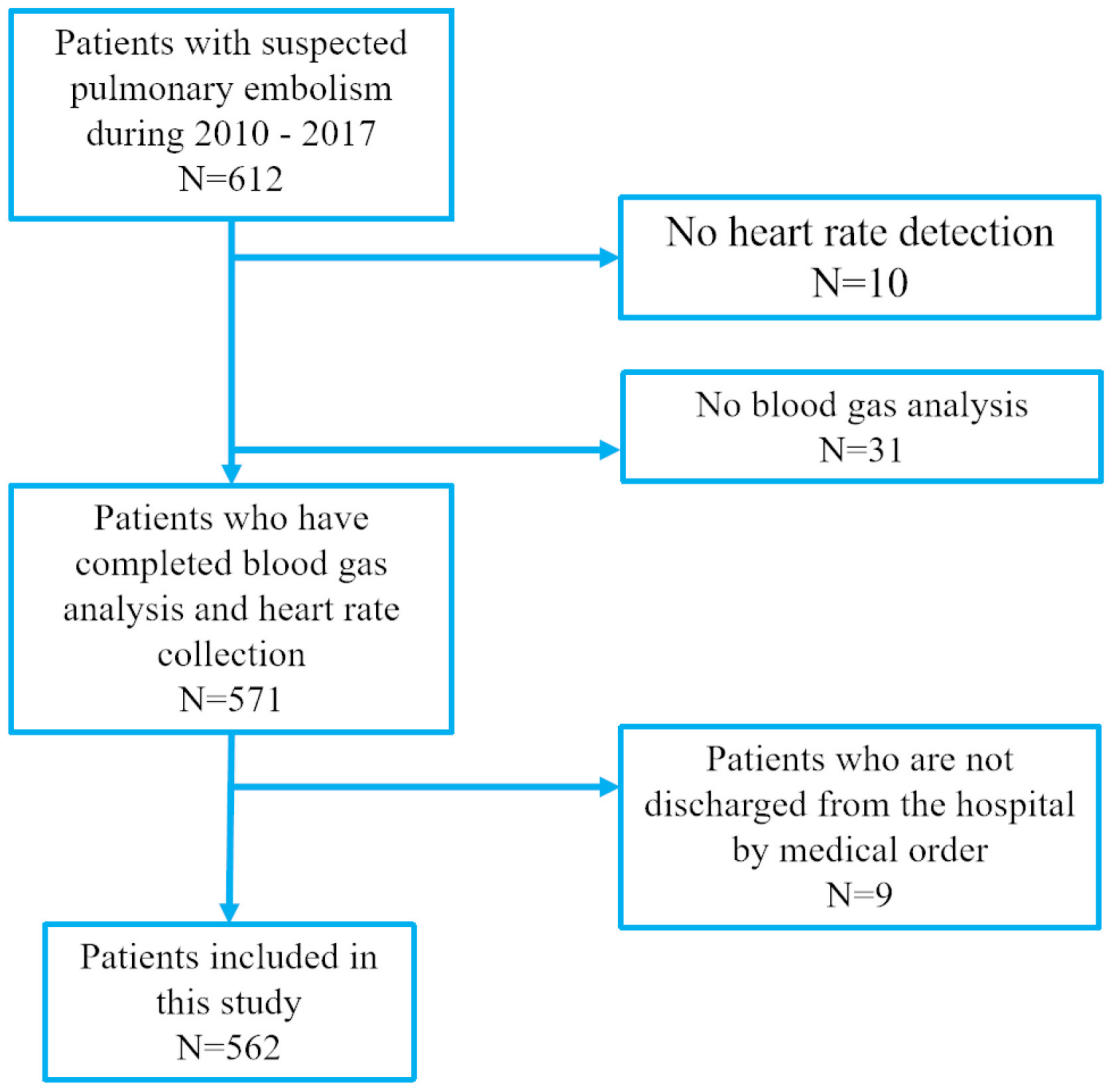
Eligibility of patients for inclusion in this study.

In accordance with the diagnostic strategy outlined in the 2019 European Society of Cardiology (ESC) Guidelines for the Diagnosis and Management of Acute Pulmonary Embolism, developed in collaboration with the European Respiratory Society (ERS), we conducted a retrospective analysis of patients admitted with pulmonary embolism (PE) to Minhang Hospital, Fudan University, between January 2010 and December 2017 (Figure 1). Cases were identified using International Classification of Diseases (ICD) discharge codes I26.0 or I26.9. For all identified patients, we reviewed electronic medical records, laboratory test results, nursing documentation, and imaging reports.

Only patients who received standardized treatment and had PE confirmed by computed tomography pulmonary angiography (CTPA) were included. For each patient, detailed baseline data were collected at admission, including demographic characteristics, medical history, presenting symptoms and signs, laboratory results, and imaging findings.

#### 2.1.1 Cohort allocation

Using a computer-generated randomization list, the study cohort was divided into two groups at an approximate 2:1 ratio: a training cohort (*n* = 373) for model development and a validation cohort (*n* = 186) for independent assessment.

#### 2.1.2 Study outcomes

The primary endpoint was all-cause mortality within 30 days. Secondary endpoints included length of recovery within 30 days (defined as hospital discharge) and 15-day mortality.

#### 2.1.3 Ethical considerations

The study was conducted in accordance with the principles of the Declaration of Helsinki and approved by the Ethics Committee of Minhang Hospital, Fudan University. The reporting followed the RECORD (REporting of studies Conducted using Observational Routinely-collected Data) statement.

#### 2.1.4 Prospective validation

Additionally, a prospective cohort of PE patients admitted to Minhang Hospital, Fudan University, between January 2024 and June 2025 was collected to validate the predictive performance of the proposed CON-PERFORM method.

### 2.2 Model development and weight optimization

Yu et al. demonstrated that the risk of adverse outcomes in pulmonary embolism (PE) is strongly correlated with patient age, heart rate, and arterial partial pressure of oxygen (PaO_2_) (19–22). Specifically, the risk is positively associated with age and heart rate, and negatively associated with PaO_2_. Based on these parameters, the PERFORM method was developed for PE risk stratification, with its scoring criteria summarized in Table 1.

**Table 1:** Scoring system based on age, heart rate, and arterial oxygen pressure.

**Variable**	**Category**	**Score**
Age (years)	< 65	0
	≥65 and < 75	1
	≥75 and < 85	2
	≥85	4
Heart rate (bpm)	< 75	0
	≥75 and < 85	1
	≥85 and < 95	2
	≥95	4
P_O2_ (mmHg)	≥80	0
	≥60 and < 80	1
	≥40 and < 60	2
	< 40	4

In the present study, we propose a novel continuous functional approach—termed **CON-PERFORM**—to quantify PE risk. The core concept is to establish a function-based model that determines a score directly derived from age, heart rate, and PaO_2_, which is used to evaluate the severity of pulmonary embolism. The assessment metric obtained through this approach exhibits good continuity. The risk evaluation function *G* is defined as:


$$\begin{array}{l} G=k_{\text{age}}\cdot \text{Age}+k_{\text{heartrate}}\cdot \text{Heartrate}+k_{{\text{PaO}}_{2}}\cdot \left(-{\text{PaO}}_{2}\right), \end{array}$$ (1)

where *k*_age_, *k*_heartrate_, *k*_PaO_2__ represent the weighting coefficients corresponding to age, heart rate, and PaO_2_, respectively. Patient-level clinical parameters are fixed and known, while the coefficients are treated as variables to be optimized. For a given triplet of coefficients, *G* can be computed for all patients. Model performance is evaluated using the receiver operating characteristic (ROC) curve, and the area under the curve (AUC) is defined as *Q*:


$$\begin{array}{r} Q=Q\left(G\right)=Q\left(G\left(k_{\text{age}},k_{\text{heartrate}},k_{{\text{PaO}}_{2}}\right)\right)\\ =Q^{\prime }\left(k_{\text{age}},k_{\text{heartrate}},k_{{\text{PaO}}_{2}}\right). \end{array}$$ (2)

This formulation treats diagnostic performance *Q* as a function of the weight coefficients. The goal is to identify the optimal set of coefficients that maximizes *Q*, thereby establishing the functional form of the CON-PERFORM method.

### 2.3 Normalization of weight coefficients

The purpose of this process is to reduce the number of independent variables and decrease the complexity of the model by analyzing the correspondence among variables. Analysis of the evaluation function reveals that scaling all coefficients by the same positive constant *N* does not affect the ROC curve or the resulting AUC, i.e.,


$$\begin{array}{l} Q^{\prime }\left(Nk_{\text{age}},Nk_{\text{heartrate}},Nk_{{\text{PaO}}_{2}}\right)=Q^{\prime }\left(k_{\text{age}},k_{\text{heartrate}},k_{{\text{PaO}}_{2}}\right). \end{array}$$ (3)

Accordingly, the coefficients were normalized such that:


$$\begin{array}{l} k_{\text{age}}+k_{\text{heartrate}}+k_{{\text{PaO}}_{2}}=1,\;k_{\text{age}},k_{\text{heartrate}},k_{{\text{PaO}}_{2}}\in \left(0,1\right). \end{array}$$ (4)

Let the relative weight ratio between heart rate and PaO_2_ be defined as *k*_heartrate_PaO_2__∈(0, 1). Then:


$$\begin{array}{l} k_{\text{heartrate}}=\left(1-k_{\text{age}}\right)\cdot k_{{\text{heartrate\_PaO}}_{2}},\\ \;k_{{\text{PaO}}_{2}}=\left(1-k_{\text{age}}\right)\cdot \left(1-k_{{\text{heartrate\_PaO}}_{2}}\right). \end{array}$$ (5)

Substituting into the evaluation function yields:


$$\begin{array}{l} G=k_{\text{age}}\cdot \text{Age}+\left(1-k_{\text{age}}\right)\cdot k_{{\text{heartrate\_PaO}}_{2}}\cdot \text{Heartrate}\\ \;+\left(1-k_{\text{age}}\right)\left(1-k_{{\text{heartrate\_PaO}}_{2}}\right)\left(-{\text{PaO}}_{2}\right), \end{array}$$ (6)

or equivalently,


$$\begin{array}{l} G=G^{\prime }\left(k_{\text{age}},k_{{\text{heartrate\_PaO}}_{2}}\right),\\ Q=Q\left(G\right)=\tilde{Q}\left(k_{\text{age}},k_{{\text{heartrate\_PaO}}_{2}}\right). \end{array}$$ (7)

This transformation reduces the number of independent variables from three to two, simplifying the optimization process and enabling three-dimensional visualization of the performance landscape.

### 2.4 Optimization procedure

Random seeds were used to generate 5,000 pairs of (*k*_age_, *k*_heartrate_PaO_2__) uniformly distributed in the range (0, 1). For each pair, the corresponding *G* values were computed across all patients, ROC curves were plotted, and the AUC (*Q*) was determined. A polynomial fitting function $\tilde{Q}\left(k_{\text{age}},k_{{\text{heartrate\_PaO}}_{2}}\right)$ was then constructed. The maximum of this function within the defined domain [*k*_age_∈(0, 1), *k*_heartrate_PaO_2__∈(0, 1)] was identified by comparing local maxima with boundary conditions. The solution provided the optimal coefficient set and corresponding maximum diagnostic performance.

Through these procedures, the CON-PERFORM score, determined by age, heart rate, and PaO_2_, can be obtained, which provides a quantitative description of the severity of pulmonary embolism.

### 2.5 Validation of diagnostic accuracy

To assess the validity of the proposed method, the evaluation function *G* = *G*(*k*_age_, *k*_heartrate_, *k*_PaO_2__) derived from the training cohort was applied to the independent validation cohort. Receiver operating characteristic (ROC) curves were plotted, and the area under the curve (AUC) was calculated. In addition, diagnostic accuracy, sensitivity, and specificity were determined to comprehensively evaluate the performance of the CON-PERFORM model.

For benchmarking, the diagnostic performance of CON-PERFORM was directly compared with that of the previously established PERFORM method, enabling assessment of the incremental predictive value achieved through the functional optimization framework.

## 3 Results and analysis

### 3.1 Clinical data and baseline characteristics

Between January 2010 and December 2017, a total of 609 patients were admitted to Minhang Hospital with a discharge diagnosis of pulmonary embolism (PE) based on ICD coding. Among these, 10 patients without documented heart rate measurements and 31 patients without arterial blood gas analysis were excluded. An additional 9 patients left the hospital before physician-approved discharge and were therefore not included in the final analysis. Ultimately, 559 objectively confirmed PE patients were enrolled, of whom 373 were assigned to the training cohort and 186 to the validation cohort.

During hospitalization, 43 patients died, including 29 in the training cohort and 14 in the validation cohort. Baseline characteristics of the two cohorts are summarized in Table 2. No significant differences were observed in age, sex, presenting symptoms, clinical signs, or past medical history (*P*>0.05). However, the two groups differed significantly in systolic blood pressure and surgical history.

**Table 2:** Baseline characteristics of training and validation groups.

**Characteristic**	**Training, *N* = 373**	**Validation, *N* = 186**	***p*-value**
Age	75 (14)	75 (14)	0.68
Gender			0.22
Female	172 (46%)	96 (52%)	
Male	201 (54%)	90 (48%)	
Dyspnea	31 (8.3%)	15 (8.1%)	0.92
Chest pain	42 (11%)	18 (9.7%)	0.51
Cough	209 (56%)	105 (56%)	0.92
Fever	65 (17%)	28 (15%)	0.48
Hemoptysis	9 (2.4%)	7 (3.8%)	0.37
Syncope	27 (7.2%)	18 (9.7%)	0.32
Altered consciousness	57 (15%)	30 (16%)	0.79
Unilateral lower extremity pain	8 (2.1%)	4 (2.2%)	0.99
Respiratory rate	21.04 (10.57)	20.08 (5.80)	0.16
Heart rate	85 (19)	84 (19)	0.33
Systolic blood pressure	129 (18)	132 (20)	0.042
Diastolic blood pressure	75 (12)	75 (13)	0.99
Jugular venous filling	102 (27%)	41 (22%)	0.18
Wet rhonchi of the lungs	173 (46%)	85 (46%)	0.88
Bilateral lower extremity edema	48 (13%)	23 (12%)	0.73
Asymmetric lower extremity edema	13 (3.5%)	6 (3.2%)	0.87
Tumor	11 (2.9%)	4 (2.2%)	0.78
Chronic cardiac disease	40 (11%)	22 (12%)	0.70
Chronic lung disease	26 (7.0%)	12 (6.5%)	0.82
Pulmonary embolism	17 (4.6%)	7 (3.8%)	0.66
Braking	198 (53%)	102 (55%)	0.69
Lower extremity deep vein thrombosis	19 (5.1%)	12 (6.5%)	0.51
Surgery	144 (39%)	43 (23%)	0.001
Hypertension	139 (37%)	77 (41%)	0.34
Diabetes mellitus	64 (17%)	32 (17%)	0.99

### 3.2 Weight coefficients and model fitting

**Figure 2:**
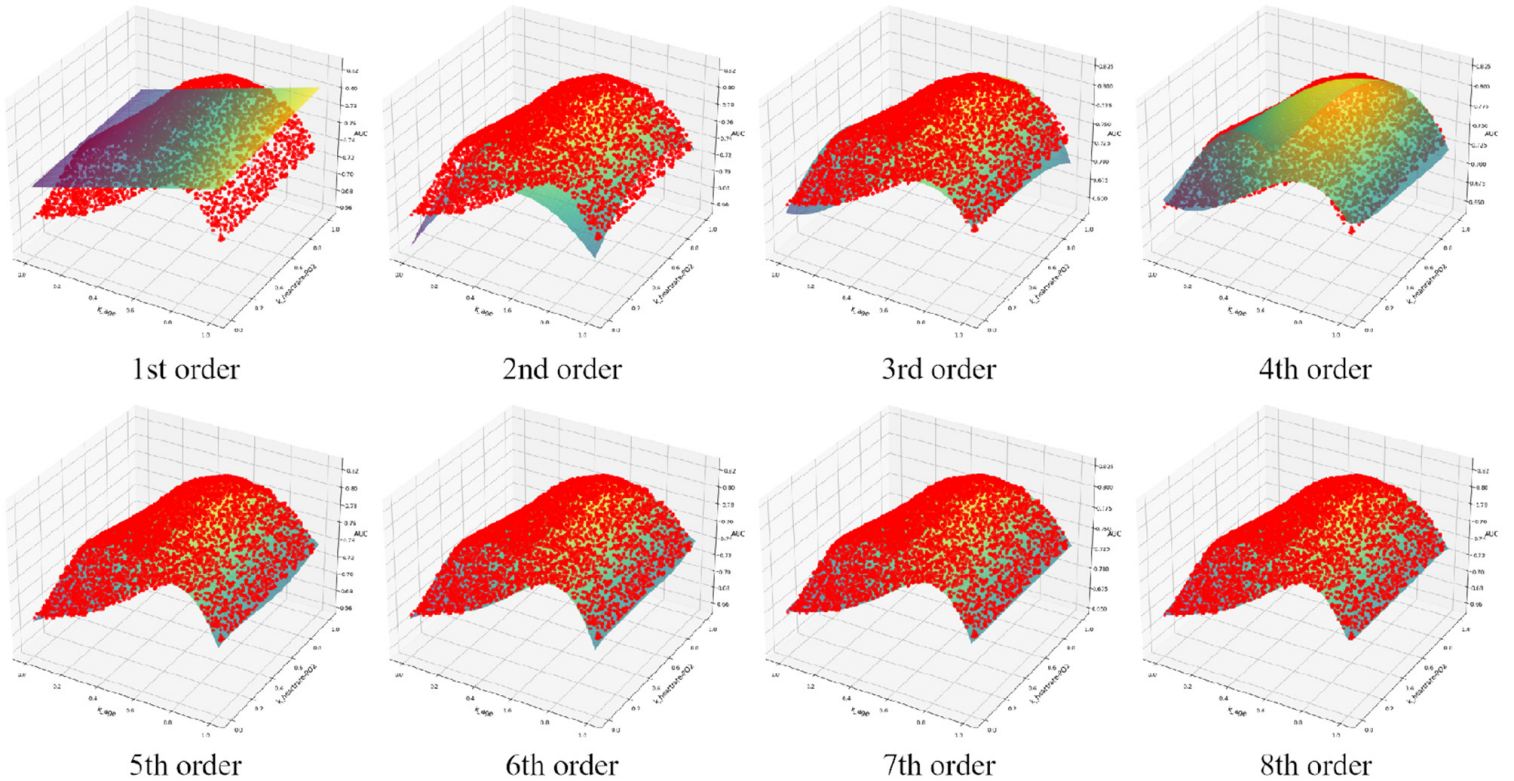
Fitting results of functions of different orders.

Polynomial fitting of the training cohort data with increasing orders of approximation is illustrated in Figure 2, and the corresponding fitting errors are summarized in Table 3. When the polynomial order reached seven, the overall fitting error dropped below 0.1%. At this stage, the fitted function was expressed as:


$$\begin{array}{cc} Q=0.7294+\left(0.0073\right)x^{1}y^{0}+\left(0.0052\right)x^{0}y^{1}+\left(0.3752\right)x^{2}y^{0}\\ +\left(1.2757\right)x^{1}y^{1}+\left(0.2045\right)x^{0}y^{2}+\left(-0.6278\right)x^{3}y^{0}+\left(-4.8399\right)x^{2}y^{1}\\ +\left(-3.8630\right)x^{1}y^{2}+\left(0.9728\right)x^{0}y^{3}+\left(-0.5804\right)x^{4}y^{0}+\left(11.5459\right)x^{3}y^{1}\\ +\left(11.0119\right)x^{2}y^{2}+\left(4.3939\right)x^{1}y^{3}+\left(-3.2441\right)x^{0}y^{4}+\left(4.1743\right)x^{5}y^{0}\\ +\left(-15.8778\right)x^{4}y^{1}+\left(-15.5061\right)x^{3}y^{2}+\left(-11.9870\right)x^{2}y^{3}+\left(1.5184\right)x^{1}y^{4}\\ +\left(1.3225\right)x^{0}y^{5}+\left(-5.4382\right)x^{6}y^{0}+\left(9.8325\right)x^{5}y^{1}+\left(11.4926\right)x^{4}y^{2}\\ +\left(8.4976\right)x^{3}y^{3}+\left(7.1073\right)x^{2}y^{4}+\left(-5.8585\right)x^{1}y^{5}+\left(2.1315\right)x^{0}y^{6}\\ +\left(2.0901\right)x^{7}y^{0}+\left(-1.9790\right)x^{6}y^{1}+\left(-2.8805\right)x^{5}y^{2}+\left(-3.9612\right)x^{4}y^{3}\\ +\left(-0.3669\right)x^{3}y^{4}+\left(-2.2781\right)x^{2}y^{5}+\left(2.7798\right)x^{1}y^{6}+\left(-1.4494\right)x^{0}y^{7} & \text{(8)} \end{array}$$

**Table 3:** Fitting errors of functions with different orders.

**Function order**	***R* ^2^**
1	0.28167
2	0.86641
3	0.98566
4	0.99243
5	0.99817
6	0.99893
7	0.99924
8	0.99936

where *x* denotes *k*_age_ and *y* denotes *k*_heartrate_PaO_2__, both ranging from 0 to 1.

Analysis of the fitted surface identified a maximum at *x* = 0.5705, *y* = 0.5945, corresponding to a function value of *Q* = 0.8405. Substituting these values yielded the optimal coefficients:


$$k_{\text{age}}=0.5705,\;k_{\text{heartrate}}=0.2553,\;k_{{\text{PaO}}_{2}}=0.1742.$$

At these optimized weights, the training cohort achieved an AUC of 0.841 (95% CI, 0.768–0.899), with a relative error of less than 0.1% compared to the polynomial-derived estimate.

### 3.3 Diagnostic performance

**Figure 3:**
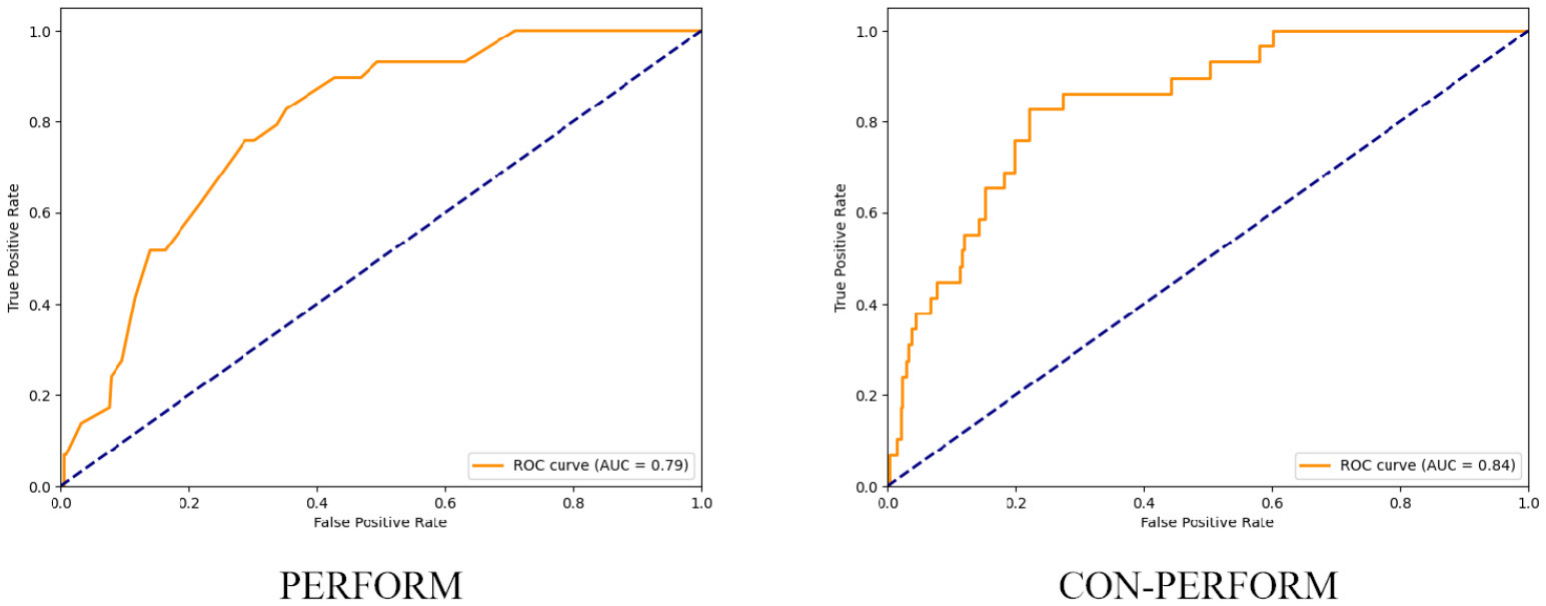
ROC curves of PERFORM method vs. CON-PERFORM method (training set).

After determining the optimal weight coefficients, receiver operating characteristic (ROC) curves of the training cohort were generated for both the PERFORM and CON-PERFORM methods (Figure 3). The corresponding diagnostic performance metrics are summarized in Table 4. The CON-PERFORM model achieved an AUC of 0.841 (95% CI, 0.776–0.902), compared with an AUC of 0.793 (95% CI, 0.719–0.856) for the PERFORM method. This represented a relative improvement of 5.1% in discriminatory ability. In addition, CON-PERFORM yielded relative increases in overall accuracy (9.4%), sensitivity (9.1%), and specificity (9.4%) compared with PERFORM. The optimal cutoff threshold for CON-PERFORM was 56.2, whereas the corresponding threshold for PERFORM was 6.

**Table 4:** Diagnostic performance of PERFORM and CON-PERFORM methods (training set).

**Metric**	**CON-PERFORM**	**PERFORM**
AUC value	0.841	0.793
Optimal threshold	56.2	6
Accuracy	0.783	0.716
Sensitivity	0.828	0.759
Specificity	0.779	0.712

**Figure 4:**
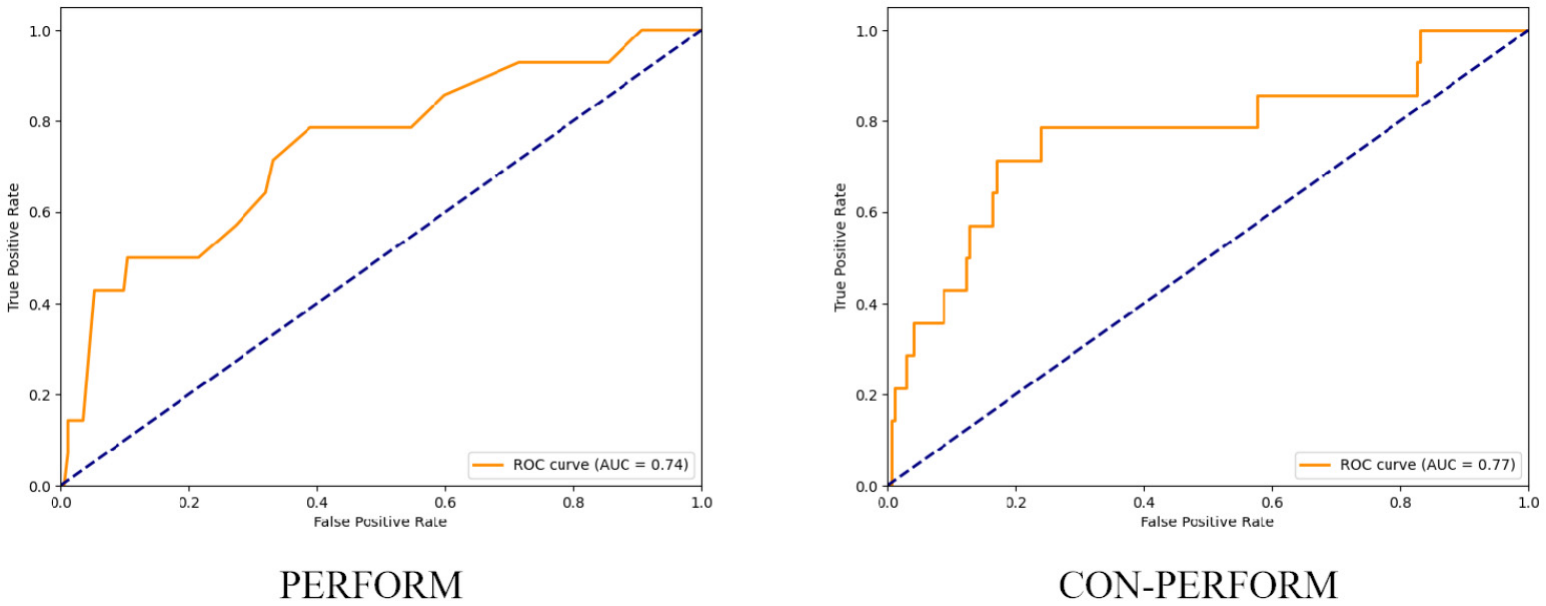
ROC curves of the PERFORM method and the CON-PERFORM method (test set).

Using the validation cohort, receiver operating characteristic (ROC) curves were generated for both the PERFORM and CON-PERFORM methods (Figure 4), and diagnostic performance metrics are presented in Table 5. The CON-PERFORM model achieved an AUC of 0.769 (95% CI, 0.607–0.916), compared with an AUC of 0.740 (95% CI, 0.579–0.880) for the PERFORM method, corresponding to a relative improvement of 3.9%. In addition, CON-PERFORM demonstrated relative increases in overall accuracy (22.4%) and specificity (24.8%), while sensitivity remained unchanged. The optimal cutoff threshold for CON-PERFORM was 54.1, representing a 3.7% shift compared with the training cohort, whereas the optimal threshold for PERFORM was 5, showing a 16.7% change relative to its training cohort threshold.

**Table 5:** Diagnostic performance of PERFORM and CON-PERFORM methods (validation set).

**Metric**	**CON-PERFORM**	**PERFORM**
AUC value	0.769	0.740
Optimal threshold	54.1	5
Accuracy	0.763	0.624
Sensitivity	0.786	0.786
Specificity	0.762	0.610

**Figure 5:**
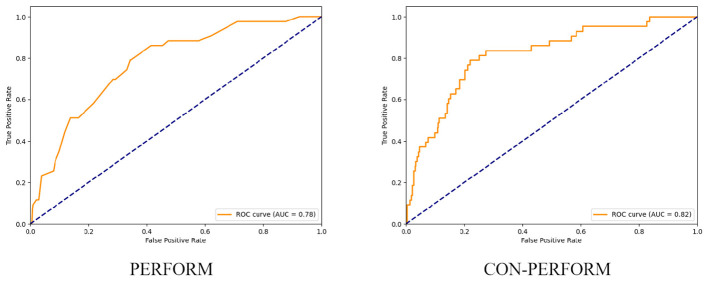
ROC curves of the PERFORM method and the CON-PERFORM method (overall data).

Using the entire study cohort, receiver operating characteristic (ROC) curves were constructed for both the PERFORM and CON-PERFORM methods (Figure 5), and diagnostic performance metrics are summarized in Table 6. The CON-PERFORM model achieved an AUC of 0.817 (95% CI, 0.730–0.876), compared with an AUC of 0.775 (95% CI, 0.705–0.838) for the PERFORM method, representing a relative improvement of 5.1%. Furthermore, CON-PERFORM demonstrated relative increases in overall accuracy (28.9%) and specificity (33.4%), while sensitivity decreased by 8.1%. The optimal cutoff threshold for CON-PERFORM was 55.6, whereas the corresponding threshold for PERFORM was 5.

**Table 6:** Diagnostic performance of PERFORM and CON-PERFORM methods (overall data).

**Metric**	**CON-PERFORM**	**PERFORM**
AUC value	0.817	0.775
Optimal threshold	55.6	5
Accuracy	0.782	0.606
Sensitivity	0.791	0.860
Specificity	0.781	0.585

### 3.4 Comparison with PESI and sPESI

Using the entire dataset, we compared the diagnostic performance of the CON-PERFORM model with PESI and sPESI. The performance metrics are summarized in Table 7. The AUC of CON-PERFORM was 0.817 (95% CI: 0.730–0.876), compared with 0.723 (95% CI: 0.654–0.787) for PESI and 0.700 (95% CI: 0.628–0.762) for sPESI. This represents a 13.0% and 16.7% increase in AUC relative to PESI and sPESI, respectively. In addition, the accuracy, sensitivity, and specificity of CON-PERFORM increased by 35.5%, -1.6%, and 40.5% compared with PESI, and by 18.1%, 21.3%, and 17.8% compared with sPESI. The optimal cutoff values were 55.6 for CON-PERFORM, 103 for PESI, and 2 for sPESI.

**Table 7:** Comparison of the diagnostic performance between COM-PERFORM and PESI/sPESI (overall data).

**Metric**	**CON-PERFORM**	**PESI**	**sPESI**
AUC	0.817	0.723	0.700
Optimal threshold	55.6	103	2
Accuracy	0.782	0.577	0.662
Sensitivity	0.791	0.804	0.652
Specificity	0.781	0.556	0.663

### 3.5 Prospective validation

To further validate the robustness of the proposed model, 128 consecutive PE cases meeting the inclusion criteria were prospectively collected at the same institution between January 2024 and June 2025. The diagnostic performance of CON-PERFORM on this prospective dataset is summarized in Table 8. The prospective cohort achieved an AUC of 0.751 (95% CI, 0.547–0.896), differing by 2.4% from the retrospective validation result. The diagnostic accuracy, sensitivity, and specificity were 0.773, 0.778, and 0.773, respectively, corresponding to relative differences of 1.4%, 1.0%, and 1.5% compared with the retrospective validation cohort. The optimal cutoff threshold was 54.6, representing a 1.0% difference relative to the retrospective validation threshold.

**Table 8:** Diagnostic performance of the CON-PERFORM method in retrospective and prospective data.

**Metric**	**Retrospective data**	**Prospective data**
AUC value	0.769	0.751
Optimal threshold	54.1	54.6
Accuracy	0.763	0.773
Sensitivity	0.786	0.778
Specificity	0.762	0.773

### 3.6 Prognostic stratification

Based on the ROC curve derived from the entire dataset, a CON-PERFORM score of 55.6 was identified as the optimal cutoff threshold. Using this cutoff, all hospitalized patients were stratified into a high-risk group (CON-PERFORM score ≥ 56.2; *n* = 146) and a low-risk group (CON-PERFORM score < 56.2; *n* = 413). Survival analysis was then performed to evaluate 30-day clinical outcomes between the two groups.

**Figure 6:**
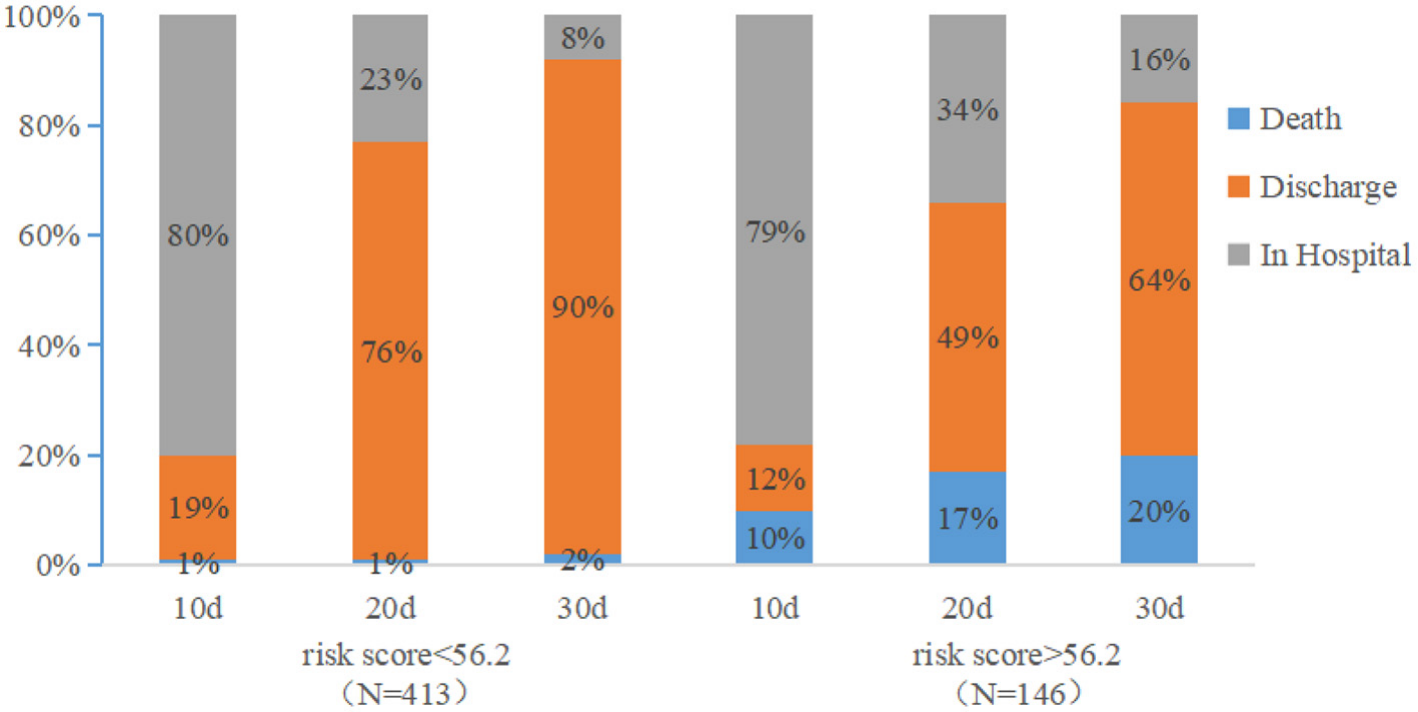
Prognostic status of patients in the low-risk group and the high-risk group at 30 days after admission.

Figure 6 illustrates the distribution of clinical outcomes (hospitalization, discharge, and death) at days 10, 20, and 30 following admission. In the low-risk group, the proportions of patients remaining hospitalized at days 10, 20, and 30 were 80%, 23%, and 8%, respectively, compared with 79%, 34%, and 16% in the high-risk group. Correspondingly, discharge rates in the low-risk group were 19%, 76%, and 90% at days 10, 20, and 30, vs. 12%, 49%, and 64% in the high-risk group. Mortality rates in the low-risk group were 1%, 1%, and 2% at days 10, 20, and 30, compared with markedly higher rates of 10%, 17%, and 20% in the high-risk group.

### 3.7 Stability analysis

In the primary analysis, 5,000 random points were generated to construct the polynomial fitting function. Table 9 presents the variation in the maximum value of the 7th-order polynomial when the number of random points was altered. As the number of sampled points increased from 1,000 to 6,000, the fluctuation in the maximum function value remained below 0.02%, indicating high stability of the fitting procedure.

**Table 9:** Maximum function values corresponding to different numbers of random points.

**Number of random points**	**Maximum value**
1,000	0.8404
2,000	0.8405
3,000	0.8405
4,000	0.8404
5,000	0.8405
6,000	0.8404

## 4 Discussion

Based on a large dataset of patients with pulmonary embolism (PE), we developed a novel functional clinical prediction method, termed the CON-PERFORM score. This model relies on only three objective variables and was shown to outperform the previously proposed PERFORM method. Unlike PERFORM, the weighting coefficients in CON-PERFORM are derived from functional optimization rather than subjective experience, and the score itself is continuous rather than categorical. This continuity allows more precise discrimination near threshold values, thereby improving overall diagnostic accuracy. For instance, under PERFORM, two patients with nearly identical physiological parameters (Patient A: age 75, heart rate 85 bpm, PaO_2_ 59 mmHg; Patient B: age 74, heart rate 84 bpm, PaO_2_ 60 mmHg) would yield markedly different scores (6 vs. 3 points). In contrast, CON-PERFORM produces continuous scores (54.21 vs. 53.21), with only a 1.8% relative difference—demonstrating its ability to maintain diagnostic precision while ensuring smoother clinical interpretation. Compared with PERFORM, CON-PERFORM demonstrated superior specificity and accuracy while maintaining comparable sensitivity.

In the CON-PERFORM model, the coefficients corresponding to age, heart rate (HR), and PaO_2_ are 0.5705, 0.2553, and 0.1742, respectively. Specifically, a one-unit increase in age has an equivalent effect to a 2.23-unit increase in HR or a 3.27-unit increase in PaO_2_ on the CON-PERFORM score. Analysis of these coefficients indicates that, for the same one-unit change, age has the greatest impact on the risk score, while PaO_2_ has the least.

Using the CON-PERFORM score, patients with PE can be stratified into high- and low-risk groups. Low-risk patients were characterized by shorter recovery times, whereas high-risk patients exhibited substantially higher mortality rates. This simple scoring method may be particularly useful in emergency departments, where it enables early identification of high-risk individuals and facilitates efficient resource allocation. With a threshold of 55.6, CON-PERFORM can identify a clinically important subgroup of low-risk patients who may be considered for outpatient management and early discharge. Conversely, patients classified as high risk (CON-PERFORM score ≥ 55.6) may warrant intensive monitoring, potentially in critical care settings (23). By relying exclusively on three objective parameters readily available at initial presentation, CON-PERFORM provides a practical, accurate, and simple prognostic model that supports individualized treatment strategies (24). CON-PERFORM provides a practical, accurate, and objective prognostic framework, representing an important step toward individualized treatment of PE. Before widespread clinical implementation, we plan to conduct a multicenter, prospective, large-sample validation study to further confirm its applicability. As you also noted, the potential of this model to reduce hospital admissions is an important future direction, as the current version was developed based on hospitalized PE patients; its utility in the general population warrants further investigation.

CON-PERFORM effectively differentiates patients with markedly distinct short-term outcomes—for instance, a 30-day mortality of approximately 20% in the high-risk group versus only 2% in the low-risk group. This substantial difference demonstrates that, beyond statistical metrics, the model provides meaningful clinical discrimination, supporting its potential utility for early risk identification and individualized management in patients with pulmonary embolism.

From the comparison between CON-PERFORM and PESI, we observed that CON-PERFORM demonstrated superior performance in AUC, accuracy, and specificity, with substantial improvements in all three metrics. The only exception was sensitivity, where CON-PERFORM was 1.6% lower than PESI. In the comparison between CON-PERFORM and sPESI, CON-PERFORM consistently outperformed sPESI across all evaluated metrics, including AUC, sensitivity, accuracy, and specificity, showing notably greater overall diagnostic performance.

At the population level, CON-PERFORM yielded higher AUC, accuracy, and specificity than PERFORM, although sensitivity was modestly reduced. This reflects the fact that the functional model was optimized for overall diagnostic performance. These findings suggest potential avenues for future research: if higher sensitivity is prioritized, alternative function analyses could be performed to construct sensitivity-optimized models.

Prospective validation further demonstrated the robustness of CON-PERFORM. The discrepancy between prospective and retrospective validation results was less than 3%, indicating high stability and generalizability, and underscoring the potential clinical utility of the model.

When the model is applied to a more heterogeneous population, a small proportion of high-risk patients could be misclassified as low-risk. We have clarified this potential limitation in the Discussion section. Importantly, our detailed subgroup analyses showed that most of these cases were near the decision boundary and did not represent clinically severe misclassifications. This observation highlights the need for further external validation in larger, multicenter cohorts to optimize the model's threshold and ensure consistent clinical safety.

In this study, the functional model includes two independent variables. Based on a power analysis assuming a moderate effect size, the minimum required sample size was estimated to be 107 cases to achieve adequate statistical power. The actual sample size used in our study exceeded this threshold, thereby meeting the basic statistical requirements for model derivation and validation.

This study has several limitations. First, as a retrospective analysis, some confirmed PE cases were excluded due to missing data, which may have introduced selection bias. Moreover, the number of deaths and the overall cohort size were relatively small, highlighting the need for larger prospective studies. Second, all cases were derived from a single institution; external validation across other hospitals or regions will be necessary to confirm the generalizability of CON-PERFORM. Third, in the current model, the weighting coefficients are treated as constants. In reality, patient-level heterogeneity may exist, and future work could explore defining coefficients as functions of patient-specific parameters to further enhance predictive accuracy. Fourth, at present, the comparison between our model and other existing methods remains insufficient. In future prospective, large-sample, multicenter studies, we plan to perform a direct comparison between COM-PERFORM and conventional indices such as PESI and sPESI. Fifth, the present study primarily examined the associations between individual parameters and all-cause mortality. In future work, we plan to further refine and expand our model to enhance its classification capability and improve its ability to meet broader clinical needs. In addition, data missingness is a common issue in clinical practice. In future studies, we plan to further explore appropriate missing-value imputation strategies and model robustness analysis methods to enhance the applicability of the model in real-world settings.

## 5 Conclusion

In this study, we developed and validated CON-PERFORM, a novel functional prognostic model for pulmonary embolism that is based on three readily available objective parameters: age, heart rate, and PaO_2_. By optimizing weighting coefficients through functional analysis, CON-PERFORM provides a continuous risk score that overcomes the subjectivity and discontinuities inherent in traditional scoring methods.

Across training, validation, and prospective cohorts, CON-PERFORM consistently demonstrated improved diagnostic accuracy, specificity, and stability compared with the PERFORM method, while maintaining comparable sensitivity. Furthermore, the score effectively stratified patients into high- and low-risk groups with distinct clinical trajectories, offering a practical tool for early risk identification and individualized management in emergency settings.

Overall, CON-PERFORM represents a simple, objective, and clinically applicable model with the potential to enhance risk stratification, guide treatment decisions, and optimize healthcare resource utilization in patients with pulmonary embolism.

## Data Availability

The raw data supporting the conclusions of this article will be made available by the authors, without undue reservation.
